# Microscale Inorganic LED Based Wireless Neural Systems for Chronic *in vivo* Optogenetics

**DOI:** 10.3389/fnins.2018.00764

**Published:** 2018-10-23

**Authors:** Raza Qazi, Choong Yeon Kim, Sang-Hyuk Byun, Jae-Woong Jeong

**Affiliations:** ^1^School of Electrical Engineering, Korea Advanced Institute of Science and Technology (KAIST), Daejeon, South Korea; ^2^Department of Electrical, Computer & Energy Engineering, University of Colorado Boulder, Boulder, CO, United States

**Keywords:** chronic, microscale LED, optogenetics, soft, wireless

## Abstract

Billions of neurons in the brain coordinate together to control trillions of highly convoluted synaptic pathways for neural signal processing. Optogenetics is an emerging technique that can dissect such complex neural circuitry with high spatiotemporal precision using light. However, conventional approaches relying on rigid and tethered optical probes cause significant tissue damage as well as disturbance with natural behavior of animals, thus preventing chronic *in vivo* optogenetics. A microscale inorganic LED (μ-ILED) is an enabling optical component that can solve these problems by facilitating direct discrete spatial targeting of neural tissue, integration with soft, ultrathin probes as well as low power wireless operation. Here we review recent state-of-the art μ-ILED integrated soft wireless optogenetic tools suitable for use in freely moving animals and discuss opportunities for future developments.

## Introduction

Precision in spatiotemporal control of a specific neuronal population is the key to dissect complex neural pathways within the nervous system. Optogenetics, a combination of optical and genetic technique, is an emerging approach that enables such highly precise, selective neuromodulation through photostimulation of genetically modified, light-sensitive target neurons. The photon sensitivity of these genetically modified cells empowers neuroscientists to study neural circuitry by stimulating or inhibiting them through exposure to different wavelengths of light (Boyden et al., 2005). Conventional tools for optogenetic studies rely on rigid and tethered silica-based optical fibers to deliver light to the deep brain from external bulky optical sources (Sparta et al., 2012), resulting in tissue damage over a relatively large volume of neural tissue. Researchers have tried to minimize tissue stress and damage by reducing device footprints in the form of small laser diode coupled SU-8 waveguides (Schwaerzle et al., 2017) and microscale inorganic light-emitting diodes (μ-ILEDs) integrated silicon probes (Wu et al., 2015; Scharf et al., 2016). However, their stiff mechanics still create unfavorable mismatch with the soft brain tissue [optical fiber: 73 GPa; silicon: 130–170 GPa vs. brain tissue: < 6 kPa (Sim et al., 2017)], which causes significant tissue lesions and inflammation over time. Recent developments helped address this issue by using biocompatible polymers to make probes flexible (Cao et al., 2013; Canales et al., 2015; Yamagiwa et al., 2015), but they still rely on tethered apparatus and fixtures with external connections which cause undue stress and constant irritation in freely moving animals by impeding their natural movement.

Recent advances in material science, micro/nanofabrication, and wireless techniques have enabled chronic, tether-free optoelectronic systems that can facilitate long-term *in vivo*optogenetics in freely moving animals. A microscale inorganic light-emitting diode is an enabling core component for such systems due to its cellular-scale form factor allowing integration onto soft, flexible probes, low power requirement making them suitable for wireless operation, and ability for precise spatial targeting of specific neurons. In this mini review article, we summarize the state-of-the-art μ-ILED integrated wireless optogenetic devices that overcome fundamental limitations of conventional rigid and tethered approaches. The article starts with discussion on requirements for an ideal neural device for chronic *in vivo* optogenetics. The following sections introduce cutting-edge soft wireless μ-ILED integrated systems in two categories – that is, head-mounted and fully implantable systems, and discuss prospects for directions of further developments to make breakthroughs in neuroscience.

## Requirements for Chronic *in Vivo* Optogenetics

**FIGURE 1:**
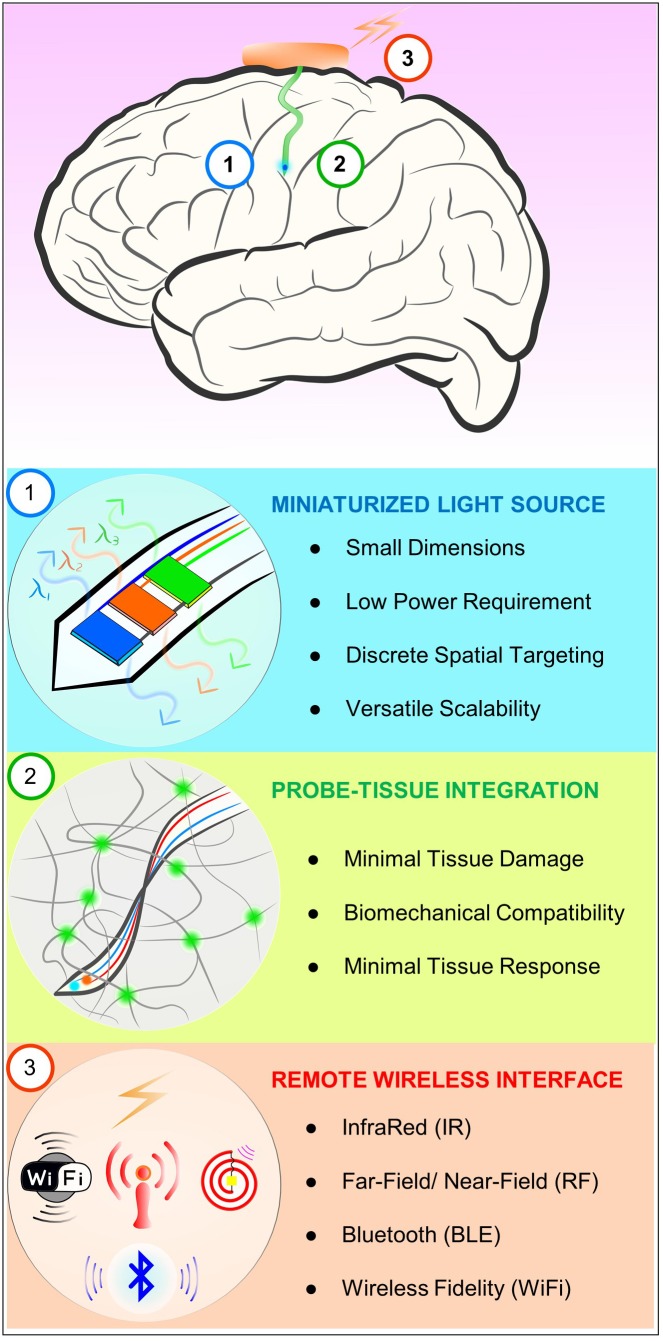
Schematic diagram illustrating desired characteristics of chronic *in vivo* optogenetic tools for freely behaving animals. Ideally, optogenetic probe systems should have miniaturized light source, biocompatible probe-tissue integration, as well as wireless control capability.

Figure 1 illustrates desired features of a neural probe system for chronic *in vivo* optogenetics. First of all, ideally, neural probe systems need a miniaturized light source. A tiny light source allows its seamless integration onto the shank of a neural probe, enabling direct light source interface with neural tissue. It can help not only reduce tissue damage and inflammation response due to its small dimensions, but also achieve more power-efficient optogenetic excitation by being right next to the target neural circuits, eliminating optical power loss associated with coupling waveguides of external light sources in the conventional waveguide-based method. Furthermore, the small size facilitates discrete, spatial targeting of a specific neuron or neural circuit for more precise, targeted optical manipulation. It can be easily extended to have multiple light sources with the same or distinct emission wavelengths on a single probe. This scalability is another beneficial attribute of miniature light sources that allow highly versatile optogenetic controls. Secondly, biocompatible probe-tissue integration should be achieved for chronic neural interface. Key factors required for chronic biocompatibility are a miniaturized neural probe for minimal tissue damage, mechanical property of the probe that matches with that of neural tissue, and material biocompatibility that causes negligible immune response. For these reasons, ultrathin, soft and flexible biocompatible polymeric platforms are considered to be an ideal solution to minimize the adverse tissue response over long periods of time. To enable chronic *in vivo* functionality while maintaining flexibility, these probes must be encapsulated with polymer with negligible water permeability (such as SU-8 and Parylene C) to provide a critical hermetic barrier against surrounding biofluids for several months (Kim et al., 2013; Shin et al., 2017). Lastly, implantable optogenetic systems should provide wireless interfaces to allow tether-free neural circuit controls to guarantee naturalistic behavior of animals. This can be realized by implementing miniaturized standalone wireless systems using widely available wireless techniques such as infrared (IR) and radiofrequency (RF) remote controls.

A μ-ILED is a core enabling element that facilitates realization of all of the above three requirements. The μ-ILEDs have dimensions smaller than 100 μm × 100 μm with the thickness of only several microns, require very low electrical input power (1–1.5 mW) for optical output intensity needed for optogenetic excitation (1 mW/mm^2^) (Kim et al., 2013), and provide various color options (e.g., blue, orange, etc.) (Park et al., 2016) as well as thermally safe operation within tissue (temperature increase of the surrounding tissue: only ∼0.1°C when operating a μ-ILED with 10 ms pulse width at 20 Hz, which produces a peak optical output power of 17.7 mW/mm^2^ at an electrical input power of 8.65 mW) (Kim et al., 2013). This favorable tiny optoelectronic component can be easily integrated with ultrathin, flexible polymeric platforms by transfer printing (McCall et al., 2013), therefore permitting minimally invasive, chronically biocompatible integration with neural tissue for *in vivo* optogenetics. For small scale integration of μ-ILEDs on flexible substrates, the polymer stamp-based transfer printing technique is favorable. Although sequential, it allows for simple and easy transfer of off-the-shelf or custom-fabricated μ-ILEDs without requiring expensive equipment and special processes, which is in contrast to wafer-level transfer using conventional laser lift-off (LLO) techniques (Delmdahl et al., 2013). LLO can allow single-step, mass transfer of custom-fabricated μ-ILEDs to other substrates, but require special thermal considerations, equipment setups, as well as careful layout design to ensure high yield and reliability for large scale transfer. Moreover, μ-ILEDs ease wireless operation due to its low operation current density requirement [ > 44 times smaller than required current density for laser coupled waveguides for optogenetic stimulation (Schwaerzle et al., 2017)], which can be easily supplied through wireless power transfer or small batteries. In comparison to laser diodes, μ-ILEDs generally operate on lower voltages and currents [2.7 V, 0.5 mA for μ-ILEDs (Jeong et al., 2015) vs. > 3 V, > 22 mA for laser diode (Schwaerzle et al., 2017)], where they demonstrate relatively higher power efficiencies and lower heat generation. Their efficiency can be further improved by limiting operational currents without affecting their capability to generate sufficient optical output power to achieve optogenetic excitation, unlike laser diodes whose threshold currents lie above tens of milliamps, making them relatively inefficient for low power wireless systems. In this short review, we discuss designs of recent μ-ILEDs integrated, soft wireless neural systems that may provide insights for future development of *in vivo* optogenetic tools.

## Soft Wireless μ-ILED Integrated Optogenetic Tools

**FIGURE 2:**
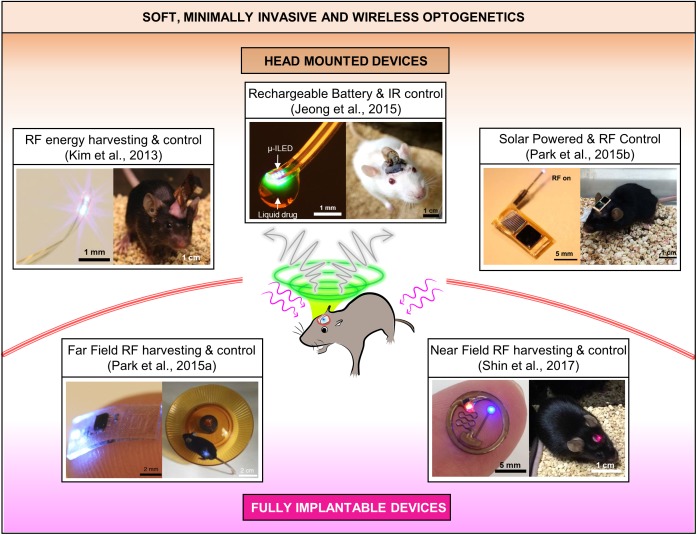
State-of-the art soft wireless μ-ILED integrated optogenetic systems, which can be grouped into head mounted and fully implantable devices. All the images are reproduced with permission from the references cited in the figure.

Combinatorial integration of μ-ILEDs based soft neural probes with different schemes of wireless controls and power supplies have created various types of soft wireless optoelectronics (Kim et al., 2013; McCall et al., 2013, 2017; Jeong et al., 2015; Park et al., 2015a,b, 2016; Shin et al., 2017; Noh et al., 2018), which have small form factors and light weights suitable for unleashed optogenetics in freely moving animals. These state-of-the art wireless neural systems allow complex behavioral studies in more natural conditions and improve chronic impact on animals by removing undue stress or irritation that can be caused by rigid implants and tethered operation. Based on implanted configurations, which mainly depend on wireless and powering schemes, the soft wireless optoelectronics can be classified into two broad categories, that is, (A) head-mounted and (B) fully implantable wireless systems, as illustrated in Figure 2 and Table 1.

**Table 1:** Comparison of the recent head-mounted and fully implantable wireless μ-ILED optogenetic tools.

Property	Head Mounted			Fully Implantable	
Reference	Kim et al., 2013; McCall et al., 2013	Jeong et al., 2015; McCall et al., 2017	Park et al., 2015b	Park et al., 2015a, 2016; Noh et al., 2018	Shin et al., 2017
μ -ILED	GaN (50 μm × 50 μm × 6.45 μm)			InGaN (220 μm × 270 μm × 50 μm)	
Probe (thickness)	PET (20 μm)	PET + PDMS (80 μm)	PI (~ 30 μm)	PDMS (80–700 μm)	PI (80–130 μm)
Encapsulation material (thickness)	SU 8 (2 μm)	SU 8 (2 μm)	SU 8 (2 μm)	Parylene C (6 μm)	Parylene C (5 μm)
Power	Wireless RF scavenging	LiPo batteries	Photovoltaic cells	RF capacitive coupling	Magnetic resonant coupling
Control (Frequency)	Far-field RF (910 MHz)	Infrared (38 kHz)	Far-field RF (1.6–2.5 GHz)	Far-field RF (1.8–3.2 GHz)	Near-field RF (13.56 MHz)
Range	~1 m	~2 m	~0.2 m	~0.2 m	~0.1 m
Weight	2 g	1.8 g	70 mg	~16–220 mg	~30 mg
SAR	0.77 mW/cm^2^	No impact	< 18 mW/kg	~ 69 mW/kg	< 20 mW/kg


### Head-Mounted Solutions

The head-mounted solutions allow highly compact, lightweight electronic systems to be exposed on the head while offering deep brain optogenetic access through soft, ultra-thin penetrating μ-ILED probes. Because the bulk of the system lies outside the body, the design can be modified with high versatility to realize diverse multi-modal platforms with less restrictions on size, powering schemes, and sensor and actuation designs. However, this approach prohibits its use in peripheral nervous systems due to relative bulkiness of the devices and is more prone to physical damage by external shocks caused during animal behavior. The following three sub-sections present head-mounted solutions based on (1) RF energy harvesting, (2) battery-powered infrared control, and (3) photovoltaic powering, respectively.

#### Injectable, Cellular-Scale Wireless Optoelectronic Systems (Kim et al., 2013; McCall et al., 2013)

Minimally invasive, standalone, soft and flexible optoelectronic probe systems were realized through transfer printing of custom fabricated gallium nitride μ-ILEDs (50 μm × 50 μm × 6.45 μm) on thin (6 μm) polyethylene terephthalate (PET) films. The cellular-scale of μ-ILEDs features highly localized targeting capabilities, minimal tissue damage and enhanced power efficiency by generating light directly near the target neurons. Owing to its design architecture utilizing transfer printing, multi-modal flexible probes can be achieved simply by stacking probes with μ-ILEDs, photodetectors (μ-IPD), and platinum (Pt) electrodes together to allow simultaneous optical modulation and electrophysiological sensing. This flexible optoelectronic probe shows reduced immune response in comparison to conventional optical fibers due to its high mechanical compliance. The design facilitates wireless control by allowing integration of detachable head-mounted RF (910 MHz) power scavenging circuits. The study demonstrated its potential for wireless optogenetics through real-time place-preference and anxiety modulation experiments in mice. Even so, the power harvested through this device is quite sensitive to directionality of the transmission panel antenna, therefore careful experimental design is necessary to cover various positions and orientations of freely moving animals during *in vivo* studies.

#### Battery-Powered, Programmable, IR Optofluidic Systems (Jeong et al., 2015; McCall et al., 2017)

To provide reliable wireless control of flexible μ-ILED probes for *in vivo* optogenetics, researchers have developed a head-mounted system that utilizes IR control, a microcontroller, and rechargeable, lightweight lithium polymer (LiPo) batteries (∼0.3 g). This IR wireless system facilitates wireless control via a simple press of button on a portable remote control, provides long range of operation (∼2 m) as well as modulation of frequency and pulse width of photostimulation in a pre-programmed way using the microcontroller. These features allow it to overcomes fundamental limitations of the RF wireless power transfer and control (Kim et al., 2013), which are significantly influenced by angles and orientations of freely behaving mice. Moreover, high discharge capabilities of the rechargeable batteries (∼80 mA) allows for integration of microfluidic pumping systems for drug delivery, which require a relative high power (on the order of hundreds of mW) for operation. Empowered by this battery-powered architecture, wireless optofluidic systems could be realized for simultaneous administration of light and pharmacological agents for highly selective, versatile manipulation of neural circuit in the brain of conscious, behaving animals. Further developments that can conquer the line-of-sight handicap of IR wireless communication and inconvenience of replacing batteries would make this technology more attractive for complex behavior neuroscience research.

#### Photovoltaic-Powered RF Wireless Optoelectronic Systems (Park et al., 2015b)

Solar cells, which harvest energy from light, are another fascinating option to power wireless optogenetic systems. To eliminate the need for rechargeable batteries for continuous operation of devices, optoelectronic systems consisting of small, lightweight gallium arsenide solar cells (5 mg), RF wireless control module and soft μ-ILEDs probes, have been developed. The wireless control was enabled by rectifying a relatively low power RF signal to manipulate a switch that connected the solar cells to the μ -ILEDs. In comparison to the same device without solar cells, this photovoltaic-assisted RF wireless optoelectronics helps increase the range of operation about three times (∼3 m) and reduce required RF power transmission levels almost by a factor of 10 (i.e., requires only 1 mW from ∼1.2 m away to realize optogenetic threshold of 1 mW/mm^2^), thus substantially decreasing electromagnetic exposure of animals. These devices also significantly minimize disturbance with animals’ free behavior by unnecessitating replacement of rechargeable batteries. However, the power reliability of solar cells can be affected by proximity, direction and nature (i.e., intensity and wavelength spectrum) of light sources in the vicinity of a freely moving mouse. Integration of solar cells with tiny chip batteries that can store back-up energy may be a potential solution to improve operation reliability.

### Fully Implantable Solutions

Advances in flexible/stretchable electronics design (Liu et al., 2016) and wireless power transfer technologies (Kim et al., 2013; McCall et al., 2013, 2017; Jeong et al., 2015; Park et al., 2015a,b, 2016; Shin et al., 2017; Noh et al., 2018) have allowed neuroscientists to implant soft optoelectronic devices completely inside the body for *in vivo* optogenetics. The compact, fully implantable soft devices guarantee more naturalistic behavior conditions to animals and provide potentials for integration with various central and peripheral nervous systems in addition to the brain. On the downside, their fully implantable nature limits their reconfigurability and functional scalability for versatile, multi-modal operation. The following sub-sections introduce fully implantable solutions based on (1) far-field and (2) near-field RF wireless energy harvesting.

#### Soft, Stretchable Optoelectronics With Far-Field RF Energy Harvester (Park et al., 2015a, 2016)

Advances in hard/soft integration techniques for electronics packaging (Liu et al., 2016) have allowed for creation of fully implantable, soft, and flexible polymeric (PDMS) implants, encapsulating μ-ILEDs and a stretchable RF energy harvester. This packaging technique not only insulates the entire device from surrounding biofluids but also provides adaptation with curvilinear surfaces of tissue. The wireless device is small (6 mm × 3.8 mm × 0.7 mm) and very light (16 mg), therefore can be subdermally implanted in various space-critical regions of both the central and peripheral nervous systems for *in vivo* optogenetics. The device can be made thinner, lighter and more flexible by further reducing the thickness of its encapsulation, thus enhancing its conformal integration with tissue. A key to miniaturization is a stretchable RF antenna design that allows capacitive coupling of ultra-high frequency (or far-field) electromagnetic waves (2.34 GHz) for energy harvesting and μ-ILED modulation. This tiny RF harvester removes the need for bulky batteries or solar cells, therefore significantly decreasing the size of the overall system. For more complicated experiments, the number of the antenna traces (i.e., operation channels) can be increased to achieve independent control of multiple μ-ILEDs at different control frequencies (Park et al., 2016; Noh et al., 2018). Wireless optogenetic excitation of spinal and peripheral nerves in live mice highlighted its versatile capabilities for *in vivo* wireless optogenetics within discrete, space-critical locations inside the body. Although showing unprecedented features, the stretchable RF antenna of this system needs design optimization to minimize the operational center frequency shift by deformation of the antenna structure caused by tissue deformation of animals in motion.

#### Flexible, Near-Field Subdermal Optoelectronics (Shin et al., 2017)

To reduce complexity in antenna designs, sensitivity to angular orientation, and RF power exposure of subjects, researchers have developed wireless subdermal flexible optoelectronics that can be powered inductively through thin coil antenna (Cu) in the near-field domain (13.56 MHz). In this device, PDMS and parylene encapsulation prevents electrical shorting of μ-ILEDs and the energy harvesting circuit due to subdermal fluid. The wireless device operated in the near-field domain considerably reduces specific absorption rate (SAR) in animal subjects compared to far-field devices as well as its dependence to surrounding environmental factors. To demonstrate their *in vivo* capabilities, dopaminergic neurons in ventral tegmental area (VTA) of mice were wirelessly triggered using the subdermally implanted device to promote their rewarding behavior. Although enabling robust wireless optogenetics in naturalistic conditions, the operation of these near-field optoelectronics require special cages installed with loop antennas parallel to the implanted optoelectronics for efficient wireless power transfer. Therefore, before experiments, care must be taken to ensure that the loop antenna and implanted devices are properly oriented each other for effective magnetic induction.

## Conclusion

Here, we review various recent soft wireless μ-ILED based optoelectronic systems that have tremendous potential for chronic optogenetics in freely behaving animals. A μ-ILED is the key to enable minimally invasive wireless optogenetic systems, which offers cellular dimensions for discrete spatial targeting of neurons and functional scalability, heterogeneous integration with flexible probes for enhanced biocompatibility, as well as power-efficient operation desired for wireless control. One of the most debated concerns regarding *in vivo* use of μ-ILEDs is the possibility of thermal damage to interfacing tissue due to their local heat generation. However, several studies have shown that this can be mitigated by controlling the output power of μ-ILEDs by limiting its operational currents and duty cycles without compromising on its ability to achieve optogenetic excitation (Kim et al., 2013; Park et al., 2015a). Moreover, tissue, working as a heat sink, facilitates fast heat dissipation, thus significantly negating the heat issue for thermally safe *in vivo* operation (Kim et al., 2013). While technologies to date focus on wireless control of neural circuitry of a specific animal, the concepts implemented here might be extended to achieve fully automated, closed loop wireless optogenetic control for high throughput neural manipulations of multiple animals. Such future development will pave the way for highly multiplexed behavioral studies to accelerate advancement of our knowledge on complex brain circuitry.

## Author Contributions

RQ and J-WJ conceived the review focus. RQ, CK, S-HB, and J-WJ reviewed literature, wrote the first draft, and finalized the manuscript. All authors approved the final version of the manuscript.

## Conflict of Interest Statement

The authors declare that the research was conducted in the absence of any commercial or financial relationships that could be construed as a potential conflict of interest.
